# Interprofessional Competence of Health and Social Care Professionals Working in Specialized Palliative Care: A Cross‐Sectional Study

**DOI:** 10.1111/scs.70241

**Published:** 2026-04-29

**Authors:** Pauliina Kesonen, Leena Salminen, Anu Soikkeli‐Jalonen, Johanna Saarinen‐Nassar, Elina Haavisto

**Affiliations:** ^1^ Department of Health Sciences (Nursing Science), Faculty of Social Sciences Tampere University Tampere Finland; ^2^ Department of Nursing Science University of Turku Turku Finland; ^3^ Turku University Hospital Turku Finland; ^4^ Tampere University Hospital Tampere Finland

**Keywords:** competence, cross‐sectional study, health‐ and social care, interprofessional care, palliative care, professionals, teamwork

## Abstract

**Aim:**

The aim of this paper is to assess interprofessional competence and explain factors associated with it among health and social care professionals working in specialized palliative care hospital wards.

**Design:**

The research study employs a descriptive cross‐sectional approach.

**Ethics:**

The study followed good scientific practice. Ethical approvals were obtained from the Ethics Committee of Tampere University (85/2022) and the Ethics Committee of the Pirkanmaa Wellbeing Services County (6/2023). Research permits were obtained separately from each institution that participated in the study. Participation was voluntary, contingent on informed consent and the confidentiality of participants was protected.

**Methods:**

In total, 153 health and social care professionals from 16 specialized palliative care wards in Finland participated in the study. The data were collected from May 2023 to March 2024, using the previously validated generic IPEC instrument and newly developed, palliative‐care‐specific ICOPA self‐assessment instruments. Data were analysed using descriptive and inferential statistics. Due to the data not being normally distributed, non‐parametric Mann–Whitney *U* and Kruskal–Wallis tests were applied, depending on the number of groups compared. Pearson's correlation was used to examine associations between variables.

**Results:**

Health and social care professionals self‐assessed their interprofessional competence as good. Among the interprofessional competencies, values and ethics were assessed highest, while teams and teamwork were assessed lowest. Education level, participation in interprofessional education, work experience, amount of teamwork conducted in their own unit, and evaluation considering teamwork functionality had a statistically significant association with professionals' better self‐assessed interprofessional competence.

**Conclusion:**

The results have identified competence gaps that will guide the development of interprofessional practice. However, because the currently used instruments are based on self‐assessment, objective measures are also needed to ensure a comprehensive evaluation of interprofessional competence.

## 1 Introduction

The aim of palliative care (PC) is to alleviate suffering and improve the quality of life of those facing life‐limiting health conditions and their families. Access to PC is considered a human right [1]. It is estimated that, worldwide, over 56.8 million people need PC each year. In Europe, the need for PC affects approximately 4.4 million people annually; it is further estimated that about 65% of those in need do not receive such care [2]. European countries vary in terms of how they provide PC services. According to studies conducted by the European Association for Palliative Care (EAPC), PC services are provided to adult patients in Europe at a rate of 0.8 per 100,000 inhabitants (median); in Finland, the rate is 0.7 per 100,000 inhabitants [3]. PC has been actively developed in Finland in recent years, reducing regional differences, improving the availability of services (including specialized PC) and enhancing the quality of palliative and end‐of‐life care at all levels [4]. To enhance care delivery, quality criteria for palliative and end‐of‐life care were drawn up, among other things to emphasize personnel education and competence and interprofessional (IP) teamwork [5].

When providing IP care, professionals are ‘working together across professions to improve equitable health outcomes’ [6]. Earlier research has shown that IP teamwork in PC is influenced by unique elements such as the need for shared decision‐making in situations of clinical uncertainty, and the necessity of balancing patient autonomy with medical judgement [7]. These factors differentiate PC from other clinical contexts, reinforcing the importance of high‐level IP competence [8, 9]. Patient‐centred and holistic care planning in PC requires an IP approach [7], drawing on the competence and teamworking of various health and social care professionals to comprehensively and individually address the care needs of patients and their family members [8]. These care needs can be physical, psychological, social, spiritual or a combination of these [1]. In specialized PC, patients and their families' care needs are even more complex [9]. In addition to physicians, nurses and social workers, the expertise of physiotherapists, spiritual care providers, psychologists, pharmacists and nutritional experts can also be utilized to meet patients' individual care needs [8]. These contextual features make IP teamwork not only desirable but essential, as no single profession can address the full spectrum of needs that patients and families experience toward the end of life [2, 6].

Professionals need to be competent in their own profession as well as in IP teamwork in order to provide effective care to patients and their family members [10, 11]. It has been recognized that PC context influence IP teamwork [8, 12] and the competence requirements of health and social care professionals [8, 13]. From the perspective of patient care, ethical aspects such as patient dignity, autonomy, respect and providing relief were found to be important [8]. Teamwork is also influenced by the patient's deteriorating condition, as the illness is no longer curable. This means that professionals must be competent in planning the patient's care in an IP manner, while also ensuring that the care does not place additional burden on the patient [12]. Although the official care relationship is with the patient, the role of family members is important in care. Family members willingly participate in their loved ones' care, also from the perspective of IP teamwork [12, 13]. As the patient's condition deteriorates, family members may increasingly require support from the IP team. Previous studies indicate that IP teamwork is not always communicated to or visible for patients and their families [12]. In addition, their role within the IP team remains unclear, even though the literature emphasizes the importance of their involvement [14]. From the professionals' perspective, team functioning is influenced by the members' ability to understand the nature and goals of PC, as well as their capacity to confront death and dying. Ethical considerations are also strongly present in interactions among professionals, appearing through respectful behaviour and ability to meet people with varying feelings [8, 15]. Additionally, certain professional qualities, such as flexibility, friendliness, patience and humility has been emphasized in PC context when working with life‐limiting health conditions [15].

A lack of competence among health and social care professionals has been identified as one of the issues with the delivery of PC [4]. Since 2011, the Interprofessional Education Collaborative (IPEC) has defined Core Competencies for Interprofessional Collaborative Practice to enhance teamwork and health outcomes. Key competencies for IP teamwork include shared values, ethical principles and mutual respect, and involve applying these in various team environments. Effective teamwork is based on responsive, responsible, respectful and compassionate communication in the team. It also involves roles and responsibilities where professionals use their own knowledge and the expertise of team members to meet patients' individual care needs [6]. To improve IP teamworking, it is important to know the type of competence professionals possess and identify areas that require further development. Competence can be assessed through various methods, and the reliability of these assessments indicates how accurately an individual's performance is measured [16]. Self‐assessment in competence assessment helps to reflect one's own performance and support professional development [17].

To our best knowledge, studies enhancing IP practices in specialized PC units are scarce. Furthermore, to date no studies have focused on the assessment of IP competence of PC health and social care professionals or how competence may differ between competence domains. This limits understanding of where targeted IP competence development is most needed. Therefore, the aim of this study is to describe and explain the IP competence of health and social care professionals working in specialized PC hospital wards. The ultimate goal is to promote the IP teamwork practices of health and social care professionals and, in turn, promote quality care for patients receiving PC and their family members.

## 2 Aim of the Study

The aim of this study is to describe and explain the IP competence of health and social care professionals working in specialized PC hospital wards.

The following research questions were formulated:

1. What kind of IP competence do health and social care professionals working in specialized PC have?
2. What factors are associated with the IP competence of health and social care professionals working in specialized PC?

## 3 Methods

### 3.1 Design

The study used a cross‐sectional design and followed the STrengthening the Reporting of OBservational studies in Epidemiology (STROBE) guidelines for cross‐sectional studies to strengthen the reporting [18].

### 3.2 Study Setting and Sample

This study was conducted in Finland, where PC is delivered following a three‐tier model: basic, specialized and advanced levels of care. At the basic level (A level), PC is provided in all health and social care units were dying people are cared for and end‐of‐life care is developed as part of daily work. Specialized PC (B level) is provided at hospital inpatient and outpatient units by hospital consulting teams and home care teams. In these units, PC is the primary focus, and staff members are more qualified in PC. Advanced PC (C level) is delivered in university hospitals' palliative centres responsible for co‐ordinating care and for the development of education and research on PC in Finland [5]. This study focuses on level B specialized hospital wards.

Health and social care professionals working in specialized PC wards were purposively invited to participate in the study to recruit a geographically representative sample. A total of 16 out of 17 wards joined the study. The inclusion criteria for participation were as follows: (1) the participant is a physician, registered nurse, practical nurse or social worker by profession; (2) the participant works in a specialized PC hospital ward; and (3) the participant is able to complete the questionnaire in Finnish.

### 3.3 Instruments

The IP competence of professionals was assessed using a generic, previously validated IPEC self‐assessment instrument (Table 1). Palliative‐care‐specific IP competence was assessed using the Interprofessional Competence in Palliative care (ICOPA) instrument (Table 2). The IPEC self‐assessment instrument consists of 42 items divided into four domains: values and ethics (*n* = 9), roles and responsibilities (*n* = 10), interprofessional communication (*n* = 11) and teams and teamwork (*n* = 12) [6]. The instrument was developed for educational purposes, but it has also been used in clinical context [19, 20]. The ICOPA self‐assessment instrument was developed for this study to complement the generic content of the IPEC instrument with more PC‐specific questions. It comprises 15 items, divided into two domains: IP teamwork (*n* = 7) and IP patient care (*n* = 8). The ICOPA instrument is based on a synthesis of previous studies in the field [8, 12, 15]. The suitability of the instruments for the PC context within the Finnish healthcare system was ensured through a literature review [8] and two qualitative studies [12, 15]. In addition, the translation process of the ICOPA instrument included content validation through expert panels, which further supported its applicability and usability in this context.

**TABLE 1:** The association between background variables and health and social care professionals' generic interprofessional competence.

Background variables	*n*	%	Mean (SD)	Values and ethics (IPEC)		Roles and responsibilities (IPEC)		Interprofessional communication (IPEC)		Teams and teamwork (IPEC)		IPEC's total competence	
				Median (Q1–Q3)	*p*	Median (Q1–Q3)	*p*	Median (Q1–Q3)	*p*	Median (Q1–Q3)	*p*	Median (Q1–Q3)	*p*
Gender	152		*na*		0.866^a^		0.478^a^		0.710^a^		0.196^a^		0.280^a^
Female	142	93.4		4.56 (4.22–4.89)		4.20 (3.90–4.75)		4.00 (3.73–4.45)		3.83 (3.50–4.17)		4.17 (3.87–4.48)	
Male	10	6.6		4.67 (4.36–4.81)		4.22 (3.93–4.43)		4.27 (3.43–4.55)		3.83 (3.04–4.10)		4.32 (3.59–4.37)	
Age (years)	152		45.4 (10.26)		0.598^b^		0.245^b^		0.245^b^		0.498^b^		0.353^b^
< 30	10	6.6		4.67 (4.47–4.81)		4.30 (4.05–4.50)		3.91 (3.77–4.23)		3.79 (3.60–4.02)		4.13 (3.95–4.30)	
30–39	36	23.7		4.67 (4.11–5.00)		4.05 (3.80–4.80)		4.00 (3.55–4.70)		3.75 (3.35–4.31)		4.17 (3.65–4.58)	
40–49	47	30.9		4.67 (4.33–4.89)		4.40 (4.00–4.70)		4.09 (3.64–4.55)		3.83 (3.67–4.25)		4.26 (3.88–4.48)	
50–59	45	28.6		4.56 (4.17–4.78)		4.20 (3.85–4.80)		4.00 (3.73–4.36)		3.92 (3.46–4.08)		4.17 (3.73–4.33)	
≥ 60	14	9.2		4.56 (4.33–4.94)		4.20 (4.00–4.80)		4.18 (3.91–4.64)		4.00 (3.67–4.42)		4.17 (3.92–4.63)	
Education	152		*na*		0.962^b^		0.062^b^		**0.029** ^**b**^		0.876^b^		0.113^b^
Vocational	44	28.9		4.56 (4.22–4.78)		4.45 (4.00–4.80)		4.00 (3.73–4.43)		4.00 (3.50–4.23)		3.23 (3.82–4.45)	
UAS	88	57.9		4.67 (4.25–4.89)		4.20 (3.90–4.60)		4.00 (3.64–4.45)		3.83 (3.52–4.17)		4.13 (3.86–4.43)	
University	20	13.2		4.67 (4.22–4.89)		4.20 (3.90–4.90)		4.41 (3.84–4.64)		3.83 (3.42–4.42)		4.21 (3.88–4.43)	
Occupation	152		*na*		0.908^b^		0.141^b^		0.280^b^		0.576^b^		0.381^b^
Practical nurse	33	21.7		4.56 (4.17–4.83)		4.50 (4.00–4.80)		4.09 (3.73–4.41)		4.00 (3.50–4.21)		4.21 (3.81–4.48)	
RN	100	65.8		4.67 (4.33–4.89)		4.25 (3.93–4.60)		4.00 (3.64–4.45)		3.83 (3.58–4.17)		4.17 (3.88–4.43)	
Physician	13	8.6		4.61 (4.06–4.89)		4.20 (3.90–4.88)		4.36 (3.89–4.64)		4.04 (3.54–4.40)		4.19 (3.95–4.67)	
Social worker	6	3.9		4.39 (4.17–5.00)		3.95 (3.50–5.00)		3.86 (3.73–5.00)		3.46 (2.98–4.04)		3.82 (3.58–4.73)	

Background variables	*n*	%	Mean (SD)	Values and Ethics (IPEC)		Roles and Responsibilities (IPEC)		Interprofessional Communication (IPEC)		Teams and Teamwork (IPEC)		IPEC's total competence	
				Median (Q1–Q3)	*p*	Median (Q1–Q3)	*p*	Median (Q1–Q3)	*p*	Median (Q1–Q3)	*p*	Median (Q1–Q3)	p
Work experience (years)													
Profession	145		16.1 (10.36)		0.285^b^		0.377^b^		**0.029** ^**b**^		**0.008** ^**b**^		0.234^b^
< 5	20	13.8		4.50 (4.14–4.78)		4.05 (3.83–4.48)		3.64 (3.36–4.28)		3.50 (3.04–3.83)		3.88 (3.51–4.30)	
5–9	31	21.4		4.67 (4.56–5.00)		4.40 (4.00–4.80)		4.27 (3.91–4.55)		4.00 (3.75–4.33)		4.29 (4.10–4.48)	
10–14	20	13.8		4.56 (4.33–4.89)		4.10 (3.80–4.40)		4.00 (3.64–4.64)		3.75 (3.50–4.17)		4.05 (3.86–4.52)	
15–19	19	13.1		4.44 (4.11–4.67)		4.00 (3.80–4.53)		3.82 (3.64–4.18)		3.67 (3.40–4.00)		3.89 (3.76–4.29)	
20–24	23	15.9		4.67 (4.33–5.00)		4.50 (4.10–4.95)		4.09 (3.77–4.50)		4.00 (3.67–4.29)		4.31 (3.98–4.62)	
≥ 25	32	22.1		4.56 (4.19–4.81)		4.20 (4.00–4.65)		4.00 (3.80–4.39)		4.00 (3.67–4.19)		4.20 (3.92–4.39)	
Palliative care	142		5.7 (4.80)		**0.037** ^**b**^		0.350 ^b^		**0.037** ^**b**^		**0.002** ^**b**^		**0.004** ^**b**^
< 5	74	52.1		4.56 (4.22–4.78)		4.20 (3.90–4.60)		3.82 (3.64–4.25)		3.75 (3.33–4.00)		4.05 (3.65–4.31)	
5–9	42	29.6		4.67 (4.56–5.00)		4.30 (4.00–4.80)		4.27 (4.00–4.55)		4.00 (3.67–4.33)		4.31 (4.07–4.52)	
10–14	17	12.0		4.61 (4.11–4.86)		4.25 (4.03–4.83)		4.05 (3.75–4.59)		4.00 (3.67–4.31)		4.13 (3.90–4.60)	
≥ 15	9	6.3		4.56 (4.14–4.94)		4.35 (3.85–4.95)		4.32 (3.73–4.45)		3.96 (3.38–4.31)		4.29 (3.74–4.58)	
Current unit	138		5.4 (4.98)		0.054^b^		0.812^b^		0.087^b^		**0.024** ^**b**^		**0.020** ^**b**^
< 5	81	58.7		4.56 (4.22–4.78)		4.20 (3.90–4.60)		3.91 (3.64–4.38)		3.75 (3.33–4.00)		4.10 (3.71–4.33)	
5–9	33	23.9		4.67 (4.56–4.97)		4.30 (4.00–4.90)		4.36 (4.00–4.70)		4.00 (3.69–4.33)		4.32 (4.10–4.63)	
10–14	12	8.7		4.61 (4.11–4.94)		4.25 (3.93–4.48)		4.00 (3.86–4.16)		4.00 (3.69–4.46)		4.10 (4.03–4.46)	
≥ 15	12	8.7		4.56 (4.14–4.94)		4.35 (3.85–4.75)		4.09 (3.73–4.43)		3.83 (3.38–4.31)		4.27 (3.74–4.54)	

Background variables	*n*	%	Mean (SD)	Values and ethics (IPEC)		Roles and responsibilities (IPEC)		Interprofessional communication (IPEC)		Teams and teamwork (IPEC)		IPEC's total competence	
				Median (Q1–Q3)	*p*	Median (Q1–Q3)	*p*	Median (Q1–Q3)	*p*	Median (Q1–Q3)	*p*	Median (Q1–Q3)	*p*
IP education	152		*na*		**0.001** ^**a**^		0.243^a^		0.065^a^		**0.008** ^**a**^		**0.004** ^**a**^
yes	48	31.6		4.78 (4.56–4.89)		4.40 (4.00–4.80)		4.27 (4.00–4.55)		4.08 (3.83–4.33)		4.31 (4.10–4.60)	
no	104	68.4		4.56 (4.22–4.78)		4.20 (3.90–4.68)		3.95 (3.64–4.36)		3.75 (3.33–4.08)		4.10 (3.77–4.36)	
Amount of teamwork	152		*na*		**0.004** ^**b**^		0.104^b^		**< 0.001** ^**b**^		**0.005** ^**b**^		**< 0.001**
Quite little	2	1.3		3.72 (3.67–3.78)		3.55 (3.30–3.85)		3.36 (3.36–3.36)		3.33 (3.17–3.50)		3.48 (3.48–3.48)	
Little nor much	5	3.3		4.22 (3.67–4.78)		3.90 (3.75–4.70)		3.73 (3.50–4.50)		3.42 (3.21–4.42)		3.71 (3.55–4.58)	
Quite much	69	45.4		4.44 (4.11–4.78)		4.10 (3.80–4.60)		3.91 (3.64–4.27)		3.75 (3.33–4.08)		4.05 (3.65–4.30)	
Very much	76	50.0		4.67 (4.44–5.00)		4.40 (4.10–4.80)		4.09 (3.82–4.64)		4.00 (3.75–4.33)		4.31 (4.05–4.60)	
Teamwork functionality	152		*na*		**0.013** ^**b**^		0.143^b^		0.064^b^		0.382^b^		0.056^b^
Quite functional	70	46.1		4.44 (4.17–4.78)		4.20 (3.90–4.60)		3.91 (3.64–4.32)		3.75 (3.38–4.08)		4.10 (3.74–4.30)	
Very functional	82	53.9		4.67 (4.44–4.89)		4.40 (4.00–4.80)		4.09 (3.73–4.50)		4.00 (3.67–4.25)		4.29 (3.90–4.57)	

*Note:* Bold values indicate statistically significant differences (*p* < 0.05).

Abbreviations: na, not applicable; Q1, quartile 25%–Q3, quartile 75%; SD, standard deviation.

^a^ Mann–Whitney *U*‐test.

^b^ Kruskall–Wallis‐test.

**TABLE 2:** The association between background variables and health and social care professionals' palliative care specific interprofessional competence.

Background variables	*n*	%	Mean (SD)	Interprofessional teamwork (ICOPA)		Interprofessional patient care (ICOPA)		ICOPA total competence		IPEC and ICOPA together	
				Median (Q1–Q3)	*p*	Median (Q1–Q3)	*p*	Median (Q1–Q3)	*p*	Median (Q1–Q3)	*p*
Gender	152		*na*		0.622^a^		0.188^a^		0.215^a^		0.183^a^
Female	142	93.4		4.29 (4.00–4.86)		4.63 (4.13–4.94)		4.47 (4.07–4.87)		4.23 (3.93–4.58)	
Male	10	6.6		4.39 (4.18–4.61)		4.75 (4.46–4.88)		4.60 (4.35–4.70)		4.39 (3.79–4.45)	
Age (years)	152		45.4 (10.26)		0.964^b^		0.608^b^		0.696^b^		0.198^b^
< 30	10	6.6		4.36 (4.11–4.79)		4.81 (4.31–5.00)		4.67 (4.27–4.85)		4.25 (4.09–4.43)	
30–39	36	23.7		4.21 (4.00–4.86)		4.56 (3.91–4.97)		4.40 (3.93–4.85)		4.22 (3.75–4.64)	
40–49	47	30.9		4.43 (4.00–4.86)		4.63 (4.25–5.00)		4.54 (4.07–4.87)		4.33 (4.00–4.61)	
50–59	45	28.6		4.29 (4.00–4.71)		4.63 (4.13–4.75)		4.33 (4.03–4.73)		4.21 (3.80–4.44)	
≥ 60	14	9.2		4.29 (4.07–4.93)		4.88 (4.31–5.00)		4.60 (4.33–4.90)		4.30 (4.04–4.70)	
Education	152		*na*		0.779^b^		0.439^b^		0.584^b^		0.445^b^
Vocational	44	28.9		4.29 (4.00–4.86)		4.50 (4.13–4.97)		4.37 (4.02–4.92)		4.25 (3.92–4.57)	
UAS	88	57.9		4.29 (4.00–4.71)		4.69 (4.13–4.88)		4.54 (4.07–4.80)		4.23 (3.88–4.52)	
University	20	13.2		4.71 (4.00–4.86)		4.63 (4.00–4.88)		4.47 (4.20–4.87)		4.26 (3.96–4.74)	
Occupation	152		*na*		0.530^b^		0.087^b^		0.532^b^		0.718^b^
Practical nurse	33	21.7		4.29 (3.93–5.00)		4.50 (4.13–4.94)		4.33 (4.00–4.97)		4.26 (3.89–4.59)	
RN	100	65.8		4.36 (4.00–4.82)		4.69 (4.13–4.97)		4.57 (4.07–4.80)		4.35 (3.91–4.52)	
Physician	13	8.6		4.14 (4.00–4.86)		4.63 (4.13–4.88)		4.43 (4.08–4.85)		4.34 (4.04–4.73)	
Social worker	6	3.9		4.79 (3.93–4.89)		3.88 (3.72–4.44)		4.30 (3.78–4.65)		3.96 (3.63–4.68)	

Background variables	*n*	%	Mean (SD)	Interprofessional teamworking (ICOPA)		Interprofessional patient care (ICOPA)		ICOPA total competence		IPEC and ICOPA together	
				Median (Q1–Q3)	*p*	Median (Q1–Q3)	*p*	Median (Q1–Q3)	*p*	Median (Q1–Q3)	*p*
Work experience (years)											
Profession	145		16.1 (10.36)		**0.041** ^**b**^		0.110^b^		**< 0.001** ^**b**^		0.487^b^
< 5	20	13.8		4.14 (3.86–4.55)		4.44 (3.91–4.63)		4.33 (3.95–4.52)		3.96 (3.68–4.35)	
5–9	31	21.4		4.71 (4.00–4.86)		4.88 (4.25–5.00)		4.80 (4.13–4.93)		4.42 (4.11–4.60)	
10–14	20	13.8		4.14 (3.86–4.71)		4.63 (4.13–4.88)		4.40 (4.00–4.73)		4.12 (3.89–4.65)	
15–19	19	13.1		4.00 (3.86–4.39)		4.19 (3.88–4.63)		4.13 (3.87–4.42)		3.96 (3.79–4.33)	
20–24	23	15.9		4.57 (4.14–5.00)		4.75 (4.19–4.88)		4.67 (4.13–4.90)		4.37 (4.07–4.69)	
≥ 25	32	22.1		4.29 (4.11–4.71)		4.75 (4.22–5.00)		4.53 (4.18–4.75)		4.22 (4.07–4.50)	
Palliative care	142		5.7 (4.80)		0.502^b^		0.160^b^		0.351^b^		0.126^b^
< 5	74	52.1		4.29 (4.00–4.71)		4.60 (4.03–4.88)		4.43 (4.00–4.80)		4.14 (3.81–4.42)	
5–9	42	29.6		4.43 (4.00–4.86)		4.75 (4.13–4.88)		4.67 (4.13–4.80)		4.39 (4.12–4.65)	
10–14	17	12.0		4.21 (4.04–4.86)		4.38 (4.03–5.00)		4.33 (4.02–4.92)		4.19 (4.00–4.69)	
≥ 15	9	6.3		4.36 (3.89–4.93)		4.56 (4.09–4.97)		4.50 (4.03–4.93)		4.34 (3.82–4.66)	
Current unit	138		5.4 (4.98)		0.278^b^		**0.019** ^**b**^		0.190^b^		0.189^b^
< 5	81	58.7		4.29 (4.00–4.86)		4.50 (4.00–4.88)		4.40 (4.00–4.80)		4.12 (3.82–4.44)	
5–9	33	23.9		4.57 (4.14–4.86)		4.88 (4.63–5.00)		4.67 (4.35–4.87)		4.39 (4.16–4.70)	
10–14	12	8.7		4.36 (4.14–4.86)		4.38 (4.16–4.97)		4.40 (4.10–4.92)		4.19 (4.10–4.54)	
≥ 15	12	8.7		4.36 (4.00–4.71)		4.50 (3.91–4.97)		4.50 (3.95–4.83)		4.34 (3.81–4.59)	

Background variables	*n*	%	Mean (SD)	Interprofessional teamworking (ICOPA)		Interprofessional patient care (ICOPA)		ICOPA total competence		IPEC and ICOPA together	
				Median (Q1–Q3)	*p*	Median (Q1–Q3)	*p*	Median (Q1–Q3)	*p*	Median (Q1–Q3)	*p*
IP education	152		*na*		**0.589** ^**a**^		**0.017** ^**a**^		**0.035** ^**a**^		**0.020** ^**a**^
yes	48	31.6		4.43 (4.14–4.86)		4.75 (4.38–5.00)		4.67 (4.33–4.93)		4.39 (4.12–4.67)	
no	104	68.4		4.29 (4.00–4.82)		4.60 (4.13–4.88)		4.40 (4.00–4.80)		4.15 (3.82–4.47)	
Amount of teamwork	152		*na*		**0.025** ^**b**^		0.136^b^		0.167^b^		**0.005** ^**b**^
Quite little	2	1.3		3.57 (3.29–3.86)		4.13 (4.13–4.13)		3.87 (3.73–4.43)		3.58 (3.54–3.61)	
Little nor much	5	3.3		4.14 (3.57–4.71)		4.13 (3.75–4.94)		4.13 (3.67–4.83)		3.82 (3.58–4.65)	
Quite much	69	45.4		4.29 (4.00–4.71)		4.63 (4.00–4.88)		4.40 (4.00–4.77)		4.14 (3.76–4.41)	
Very much	76	50.0		4.57 (4.14–4.86)		4.75 (4.25–5.00)		4.67 (4.13–4.87)		4.39 (4.11–4.67)	
Teamwork functionality	152		*na*		0.082^b^		0.378^b^		0.504^b^		**0.005** ^**b**^
Quite functional	70	46.1		4.29 (4.00–4.64)		4.63 (4.00–4.88)		4.47 (4.00–4.70)		4.18 (3.82–4.42)	
Very functional	82	53.9		4.29 (4.00–4.86)		4.63 (4.13–5.00)		4.67 (4.13–4.87)		4.33 (4.01–4.62)	

*Note:* Bold values indicate statistically significant differences (*p* < 0.05).

Abbreviations: na, not applicable; Q1, quartile 25%–Q3, quartile 75%; SD, standard deviation.

^a^ Mann–Whitney *U*‐test.

^b^ Kruskall–Wallis‐test.

Both instruments utilized a five‐point Likert‐type scale for responses, from ‘strongly disagree’ [1] to ‘strongly agree’ [5], with a higher total score indicating higher self‐assessed competence. The items were presented in the form ‘I am able to’. Additionally, background information was collected. Background factors (*n* = 12) comprised age, gender, level of education, profession, working experience in the profession, working experience in PC, working experience in the current unit and participation in IP education. Furthermore, professionals were asked to assess whether IP teamwork occurs in their unit (yes/no), to assess the amount of IP teamwork on a five‐point Likert scale (from ‘quite little’ to ‘very much’), and to assess the functionality of teamwork on a five‐point Likert scale (from ‘not functional at all’ to ‘very functional’).

### 3.4 Data Collection

The data were collected electronically from May 2023 to March 2024 using a Microsoft Forms e‐questionnaire. An online survey was selected due to the long geographical distances and cost‐effectiveness of this method of data collection [21]. A total of 394 professionals were recruited to participate in the study. The survey was sent electronically to 369 health and social care professionals, and 25 hard‐copy questionnaires were distributed at the request of one participating ward. Paper‐and‐pencil questionnaires were provided at the request of respondents and returned in sealed envelopes.

After receiving the research permits and before starting the data collection, the research team either visited staff or organized an online meeting to inform them about the ongoing study. Staff members then had the opportunity to ask questions. Each participating ward was asked to designate a contact person. The contact person emailed the questionnaire to all professionals working in the unit. Emails were sent a total of three times; two reminders were sent every 3 weeks. The emails contained information about the ongoing study, its aim and purpose, the voluntary nature of participation, data protection and contact information for further questions. Before completing the questionnaire, participants were asked to give their informed consent to participate in the study; without written consent, access to the questionnaire was denied.

### 3.5 Data Analysis

The software SPSS Version 29.0.20 [26] for Windows was used for the data analysis. Descriptive and explanatory statistics were used to summarize the data. Because the data were abnormally distributed with high skewness, nonparametric tests were applied. Medians and quartiles were used to describe the data. The participants' demographics were described using means, frequencies, percentages and ranges where applicable. Missing values in the background data were described by reporting the *n*‐values. The sum variables represent the domains of each instrument. The IPEC instrument has four sum variables, and the ICOPA instrument consists of two sum variables based on the exploratory factor analysis (EFA). Regarding the sum variables, at least two thirds of the items had to be answered for a variable to be included in the analysis. In fact, only a few individual values were missing.

Professionals' IP competence was described using medians, lower quartiles and upper quartiles, means and standard deviations. The association between background variables and self‐assessed IP competence was examined using the Mann–Whitney test. The Kruskal–Wallis test with Bonferroni correction was used to describe categorical variables, and Spearman's correlation coefficients were used for continuous variables. The threshold for statistical significance was set at *p* < 0.05 [22]. The results are presented using *p*‐values, medians and quartiles.

## 4 Results

### 4.1 Participant Characteristics

A total of 153 professionals from 16 specialized PC wards completed the questionnaire; 137 participants completed the electronic questionnaire and 16 completed it on paper, representing an overall response rate of 38.8%. Most of the participants were female (92.8%), and the mean age of participants was 45.4 years, with ages ranging from 23 years to 65 years. Of the participants, 70.6% had higher education (university or university of applied sciences). Average overall work experience was 16.1 years (ranging from 0.2 to 42.8 years), time in PC was 5.7 years (ranging from 0/recently started to 22 years) and time in the current unit was 5.4 years (ranging from 0.1 to 21.0 years) (see Table 3).

**TABLE 3:** Professionals' education and work experience in years.

Profession (*n* = 153)	Registered nurse (*n* = 100, 65.4%)	Practical nurse (*n* = 33, 21.6%)	Physician (*n* = 13, 8.5%)	Social worker s(*n* = 63, 9%)
Education^a^				
Upper secondary (vocational)	14	30	0	0
University of Applied Sciences (UAS)	84	< 5	0	< 5
University	< 5	< 5	13	4
Work experience				
Current profession				
≤ 5	17	2	0	1
5–9	22	6	2	1
10–14	12	4	4	0
15–19	11	6	1	1
20–24	14	7	2	0
≥ 25	21	4	4	3
Palliative care				
≤ 5	50	16	4	4
5–9	30	8	4	0
10–14	11	3	2	1
≥ 15	5	2	1	1
Current unit				
≤ 5	57	15	5	4
5–9	23	4	5	1
10–14	7	4	1	0
≥ 15	7	4	0	1

^a^ Information missing.

About a third of the professionals (31.4%) had participated in IP education. All professionals reported that they work interprofessionally within their unit: 4.6% (*n* = 7) of participants reported that they work interprofessionally ‘quite little’ or ‘neither little nor much’, but the majority of participants (94.8%, *n* = 145) reported that they work interprofessionally either ‘quite a lot’ or ‘very much’ in their unit. Altogether 98.7% of respondents indicated that IP teamwork in their unit was ‘quite or very functional’.

### 4.2 Professionals Self‐Assessed Interprofessional Competence in Specialized Palliative Care

Participants assessed their total IP competence (Table 4) as good, generic IP competence (IPEC) (median 4.17, quartiles 3.87–4.48) as high, but PC‐specific IP competence (ICOPA) as slightly higher (median 4.53, quartiles 4.07–4.80). Regarding the domains, the highest competence was reported in values and ethics (IPEC; median 4.56, 4.22–4.89) and IP patient care (ICOPA; median 4.63, 4.13–4.88). The lowest competence was reported in teams and teamwork (IPEC; median 3.83, 3.50–4.17).

**TABLE 4:** Professionals' self‐perceived interprofessional competence in specialized palliative care (*n* = 152–153).

Competence domain	Median (Q1–Q3)
Generic IPEC total	4.17 (3.87–4.48)
IPEC values and ethics	4.56 (4.22–4.89)
IPEC roles and responsibilities	4.30 (3.90–4.70)
IPEC communication	4.00 (3.73–4.45)
IPEC teams and teamwork	3.83 (3.50–4.17)
PC‐specific ICOPA total	4.53 (4.07–4.80)
ICOPA IP teamwork	4.29 (4.00–4.86)
ICOPA IP patient care	4.63 (4.13–4.88)

### 4.3 The Association Between Background Factors and Interprofessional Competence

The following background factors had a statistically significant association with health and social care professionals' self‐assessed IP competence: higher‐level education, participation in IP education, work experience in the profession, work experience in PC and work experience in the current unit. In addition, an association was found between professionals' experience of IP teamwork frequency and functionality.

Professionals with a higher‐level education rated their generic IP communication competence higher than others (Md 4.41, 3.84–4.64). Participation in IP education at any point in participants' training or career was significantly associated with higher total IP competence (*p* = 0.020), generic IP competence (*p* = 0.004), values and ethics (*p* = 0.001) and teams and teamwork competence (*p* = 0.008). Participants who reported frequent IP work in their unit rated their IP competence higher in all areas except roles and responsibilities. While evaluations of teamwork functionality were initially associated with total IP competence (*p* = 0.005) and values and ethics (*p* = 0.013), no pairwise differences remained significant after Bonferroni correction. Participants work experience in their profession was significantly associated with IP communication (*p* = 0.0029) and teams and teamwork (*p* = 0.006); experienced professionals rated their competence higher than early‐career professionals. Years of experience in PC were significantly associated with total generic IP competence (*p* = 0.004), values and ethics (*p* = 0.026), IP communication (*p* = 0.037) and teams and teamwork (*p* = 0.002); a notable increase in competence was observed among those with 5 to 10 years of experience compared to early‐career professionals. Additionally, years of work experience in the current unit were associated with total generic IP competence (*p* = 0.020) and teams and teamwork (*p* = 0.024), suggesting competence growth in these areas (see Table 1).

Participation in IP education, either during professional education or later in participants' careers, was significantly associated with higher total PC‐specific IP competence (*p* = 0.035) and IP patient care competence (*p* = 0.017). While the amount of IP teamwork was initially associated with PC‐related IP teamwork competence, no pairwise differences remained significant after Bonferroni correction. A similar pattern was observed for years of professional experience and IP teamwork competence. Overall, self‐assessed total IP competence in PC varied with years of experience, showing an increase in early career (< 5 to 5–10 years), a decline around 15–20 years and a subsequent increase after 20 years of practice (see Table 2).

### 4.4 Ethical Considerations

This study adhered to the principles of the Declaration of Helsinki [23]. Ethical approval was requested and received from the Ethics Committee of Tampere University (85/2022) and the Ethics Committee of the Pirkanmaa Wellbeing Services County (6/2023). Also, permissions were obtained from all the organizations from which data were collected. The European Union's data protection legislation, for example the General Data Protection Regulation 2016/679, was adhered to during data collection [24]. Permission to translate the IPEC tool into Finnish, use it for data collection and publish the results was sought and obtained via email (4.1.2023) from the owner of the tool. The use of the ICOPA instrument did not require any permissions, as it was developed by the research team.

Participants received information about the research study both verbally and in writing. They were informed about the study in an email that outlined the purpose of the study, the method of data collection, details about the preservation of the data and subsequent use, anonymity and the voluntary nature of participation—participants had the right to withdraw at any stage of the study but not after the analysis had been conducted. The researchers' contact information was provided for further questions. Because of the small number of suitable professionals, only implicit personal data were collected. Before beginning the survey, participants were directed to an information page asking participants for their informed consent. Only those who answered ‘yes’ to the question were given access to the questionnaire [25].

## 5 Discussion

This study aimed to describe and explain the IP competence of health and social care professionals working in specialized PC hospital wards in Finland. The complex care needs of patients, and their family members make IP teamwork and related competence particularly important in this care context [9]. To provide high‐quality care for patients and their families, competence in both one's own profession and IP teamwork are essential [10, 11]. To gain a comprehensive understanding of IP competence in PC context, the generic IPEC competence instrument [6] was combined with the PC‐specific ICOPA instrument [8, 12, 15] developed for this study. Health and social care professionals who participated in direct patient care and daily clinical decision‐making in specialized PC wards were involved.

Overall, health and social care professionals assessed their IP competence as good; only a few statistically significant differences were observed. The highest self‐assessed competence was found in ethically highlighted areas such as reflecting professionals' ability to prioritize patient‐centred care, follow ethical standards and support patients with life‐limiting health conditions and their family members. This finding is understandable in light of the care context, as specialized PC units rely heavily on IP teamwork as a core mechanism for delivering holistic, patient‐ and family‐centred care [1, 7, 9], and professionals in these settings engage in continuous teamwork with multiple professions [8, 9]. Because high‐quality PC requires integrating physical, psychological, social and spiritual perspectives into care planning [1, 7, 8], professionals—particularly nurses—gain extensive, practice‐embedded exposure to IP work [8, 9]. This may strengthen their perceived competence over time and contribute to the consistently high self‐assessment observed in our study.

The stronger assessment on ethical areas is consistent with previous research emphasizing the importance and presence of ethical aspects in PC [8, 15]. This may be resulted because professionals who are particularly interested in existential, ethical and sensitive aspects of care are more likely willing to work in PC. Professionals attracted to PC care values dignity, autonomy, and relief of suffering, and where unit cultures explicitly reinforce constant consideration of ethical values. Practices such as care planning, and attention to goals‐of‐care discussions and decision‐making processes may also strengthen perceived competence in these domains [8, 12, 15]. By contrast, competence in teams and teamwork and IP communication was rated lower, indicating areas requiring further development. The importance of communication skills in both IP teamwork and patient care is undeniable [6], and due to the care context, the issues being communicated are often highly sensitive. As post‐qualification education tends to focus primarily on patient care and development of clinical skills, it is important to support IP communication through training but also other means. It has been suggested that less formal approaches, such as shared activities outside of work, may at best strengthen relationships among professionals and thereby facilitate communication within the IP team [26].

Research on IP competence and its associated factors remains limited in PC, making it challenging to fully understand professionals' competence in this context. The ‘wave‐like’ pattern observed in how professionals' IP competence self‐assessment varied over the course of their working years may reflect changes in self‐awareness and professional roles over time. It can be seen, that early‐career professionals may lack confidence, mid‐career professionals may assess their abilities more critically, and in their later career, professionals may have more confidence due to experience. Participation in IP education appears to enhance competence by exposing professionals to collaborative learning and sharing their professional perspectives [5]. Such experiences strengthen communication and teamwork skills, which are transferable to clinical practice [20]. Similarly, accumulated experience and longer tenure within the same unit foster team familiarity, trust and shared understanding, which support better teamwork in patient care.

The constitution of the IP team deserves closer examination in the future. According to current recommendations, IP teamwork is encouraged to support patients' overall quality of life; however, teamwork appears to be concentrated among certain professional groups. Teamwork dynamics and interaction within the teams should be examined at both the team and organizational levels in order to enable broader and more inclusive IP teamwork. Specialized PC teams can include a wide range of professionals [8]. In PC, maintaining quality of life and avoiding unnecessary medical interventions are emphasized as key elements of care [1]. Nevertheless, care still appears to be predominantly physician‐ and nurse‐led [8], which was also reflected in the study population of this study. This study focused deliberately on registered and practical nurses, physicians and social workers to ensure participant anonymity, considering the small number of professionals working in specialized PC in Finland. Moreover, in these units, certain professionals, for example physiotherapists and occupational therapists, tend to contribute mainly through consultations instead of being fully integrated into the IP team. This influences their assessment of IP teamwork and related competencies. Further research should include less represented professional groups to gain a more comprehensive understanding of IP competence across PC context and across organizational boundaries as it could help to further develop the service system and, consequently, improve the care and quality of life of patients receiving PC. Moreover, the patient and family members' role in an IP team remains somewhat unclear further research is needed to support their involvement so that they are not viewed merely as recipients of care but as active members of the IP team.

To assess professionals' competence, this study relied on self‐assessment instruments, which may have led to participants overestimating their IP competence. The use of self‐assessment instruments in evaluating competence is one of the key prerequisites for professional development [27], however, to enable a more comprehensive assessment of competence, future research should therefore incorporate objective measures to complement these findings. Participants of the study were qualified professionals; competencies required for teamwork should be strengthened also after graduation, which highlights the need for organizational support for effective IP teamwork. However, participants reported strong PC‐specific teamwork competencies, such as flexibility, collegiality, compromise and mutual support, which may be partly explained by the fact that participants are more educated in PC and based on working years were experienced in the field. In PC, IP teamwork is an integral part of patient care. Close collaboration between various health and social care professionals and patients' specific care situations may promote the development of IP competence as part of daily practice.

### 5.1 Strengths and Limitations

While the study has several strengths, certain limitations should also be acknowledged. The study was based on geographically representative data from across Finland. However, caution should be exercised when transferring the findings to other PC contexts, as service systems may differ culturally and between different levels of care. Using purposive sampling method enabled collecting data from units where PC is the main focus. Although the response rate of 38.8% can be considered good for an online survey, a higher response rate would have strengthened the representativeness of the data. Nurses, physicians and social workers completed the questionnaire. The participants were mainly from a nursing background, but the distribution is similar to the typical structure in the Finnish service system. Although all specialized PC wards in Finland were recruited for the study, the overall population remains small for a survey. Some professional groups within the IP team were represented by only a few individuals, but their role in the team is undeniable. However, to ensure anonymity, these groups were therefore not included in the study. Self‐assessment represents only one aspect of competence evaluation. As professionals may overestimate their own competence [27], the results should be interpreted with caution. Future studies should complement self‐assessment with objective assessments where possible. Neither the Finnish translated IPEC tool nor the ICOPA instrument were pilot‐tested before the data collection [28]. Psychometric testing is needed to examine them in more detail. Cronbach's *α* values were examined at the instrument and domain levels, and they ranged from 0.870 to 0.966 (see Table 5).

**TABLE 5:** Cronbach's alpha values for the IPEC and ICOPA instruments and their domains.

Instrument/domain	Number of items	Cronbach's α
IPEC	42	0.966
(1) Values and ethics	9	0.870
(2) Roles and responsibilities	10	0.905
(3) Interprofessional communication	11	0.905
(4) Teams and teamwork	12	0.919
ICOPA	15	0.918
(1) IP patient care	8	0.879
(2) IP teamwork	7	0.856

## 6 Conclusion

Health and social care professionals working in specialized PC indicated good self‐assessed IP competence. According to their self‐assessments, their strongest competences were related to values and ethics as well as IP teamwork in patient care, whereas the weakest areas concerned communication and generic teamwork. However, since self‐assessment instruments were used, no objective information is available on the actual level of professionals' competence. Therefore, in future the development of objective assessment tools should be considered. To better align future research on IP teamwork with current care recommendations, it is important to include patients' family members as well as less represented professionals' groups who are equally an important part of the IP team. In addition, education in IP teamwork in professional and continuing education should emphasize communication skills and the ability to work in IP teams, engaging functional teamwork across professional boundaries. This is especially important in PC, which requires close collaboration among different professional groups as well as effective communication with patients and families to ensure high‐quality care.

## Author Contributions

Study design: P.K., L.S., E.H. Data collection: P.K., E.H., A.S.‐J., J.S.‐N. Data analysis: P.K., L.S., E.H. Manuscript writing: P.K. Critical revision of the article: L.S., E.H., A.S.‐J., J.S.‐N.

## Funding

The study was partially funded through The Finnish Association of Nursing Research (HTTS).

## Conflicts of Interest

The authors declare no conflicts of interest.

## Data Availability

The data is not publicly available due to privacy or ethical restrictions.
